# Angiotensin converting enzyme 2 gene expression and markers of oxidative stress are correlated with disease severity in patients with COVID-19

**DOI:** 10.1007/s11033-023-08515-0

**Published:** 2023-05-24

**Authors:** Afraa S. H. Alobaidy, Mona Elhelaly, Maggie E. Amer, Rasha S. Shemies, Azza I. Othman, Mohamed A. El-Missiry

**Affiliations:** 1 Zoology Department, Faculty of Science, Mansoura University, Mansoura, Egypt; 2 Medical Biochemistry and Molecular Biology Department, Faculty of Medicine, Mansoura University, Mansoura, Egypt; 3 Mansoura Nephrology and Dialysis Unit, Faculty of Medicine, Mansoura University, Mansoura, Egypt

**Keywords:** Angiotensin converting enzyme 2 gene, Melatonin, Malondialdehyde, Total antioxidant capacity, COVID-19, SARS-CoV-2

## Abstract

**Background:**

Oxidative stress is thought to play a significant role in the pathogenesis and severity of COVID-19. Additionally, angiotensin converting enzyme 2 (ACE2) expression may predict the severity and clinical course of COVID-19. Accordingly, the aim of the present study was to evaluate the association of oxidative stress and ACE2 expression with the clinical severity in patients with COVID-19.

**Methods and results:**

The present study comprised 40 patients with COVID-19 and 40 matched healthy controls, recruited between September 2021 and March 2022. ACE 2 expression levels were measured using Hera plus SYBR Green qPCR kits with GAPDH used as an internal control. Serum melatonin (MLT) levels, serum malondialdehyde (MDA) levels, and total antioxidant capacity (TAC) were estimated using ELISA. The correlations between the levels of the studied markers and clinical indicators of disease severity were evaluated. Significantly, lower expression of ACE2 was observed in COVID-19 patients compared to controls. Patients with COVID-19 had lower serum levels of TAC and MLT but higher serum levels of MDA compared to normal controls. Serum MDA levels were correlated with diastolic blood pressure (DBP), Glasgow coma scale (GCS) scores, and serum potassium levels. Serum MLT levels were positively correlated with DBP, mean arterial pressure (MAP), respiratory rate, and serum potassium levels. TAC was correlated with GCS, mean platelet volume, and serum creatinine levels. Serum MLT levels were significantly lower in patients treated with remdesivir and inotropes. Receiver operating characteristic curve analysis demonstrates that all markers had utility in discriminating COVID-19 patients from healthy controls.

**Conclusions:**

Increased oxidative stress and increased ACE2 expression were correlated with disease severity and poor outcomes in hospitalized patients with COVID-19 in the present study. Melatonin supplementation may provide a utility as an adjuvant therapy in decreasing disease severity and death in COVID-19 patients.

## Introduction

Coronavirus disease 2019 (COVID-19), caused by severe acute respiratory syndrome coronavirus-2 (SARS-CoV-2), is a highly contagious and pathogenic viral infection that caused a global pandemic with a substantial number of deaths [1]. All coronaviruses have specific genes that code for proteins required for viral replication, nucleocapsid formation, and spike production[2]. The classic method for coronavirus entry into host cells is receptor-mediated endocytosis[3]. SARS-CoV-2 has been shown to enter the host cells through interactions with the angiotensin converting enzyme 2 (ACE2) receptor [4], with recognition of the ACE2 receptor by SARS-CoV-2 dependent on the structure of the coronavirus spike protein [5].

Interactions between the coronavirus spike protein and ACE2 causes increased expression of angiotensin II (Ang II), which in turn activates NADPH oxidase and increases oxidative stress[6]. Moreover, NADPH oxidase enhances the production of reactive oxygen species (ROS), a process regulated by the transcriptional protein nuclear factor kappa-light-chain-enhancer of activated B cells (NF-kB) [7]. The generation of ROS represents an integral component of the host defenses against invading microorganisms. Oxidative stress induced by viral infection enhances cytokine production leading to activation of innate immune responses[8]. However, excessive production of ROS induced by respiratory viral infections may contribute to lung tissue injury and damage [9]. Peroxynitrite is a powerful oxidant that is known to cause pulmonary injury and loss of pulmonary function in patients with a range of viral infections[10]. Interestingly, melatonin (MLT) is an efficient scavenger of peroxynitrite and inhibitor of nitric oxide production that has been shown to reduce lipid peroxidation during lung injury[11, 12].

Oxidative stress is postulated to be involved in the pathogenesis of COVID-19 [13]. Both the SARS-CoV-2 coronavirus and the pro-inflammatory cytokines generated during SARS-COV-2 infection, such as IL-1β, IL-2, IL-6, TNF-α, and interferon-γ (IFN-Y), predominantly target the endothelium [14, 15], thereby increasing endothelial permeability for macromolecules that may precipitate pulmonary injury [16]. In turn, inflammatory cytokines can augment oxidative stress via stimulation of neutrophils, macrophages, and endothelial cells [17]. The complex interactions between oxidative stress and inflammatory cytokines promote cytokine storm in COVID-19 patients and may thereby predict disease severity and poor outcomes [18]. Early anticipation and management of cytokine storm using immunomodulators and blood purification systems may have utility in improving the survival of patients with COVID-19 [19, 20]. Targeting oxidative stress represents a potential therapeutic option that warrants further study of the role of oxidative stress in pathogenesis of COVID-19 [21]. Accordingly, the present study aimed to assess the association of ACE2 expression and markers of oxidative stress, including malondialdehyde (MDA), total antioxidant capacity (TAC), and melatonin (MLT), with the clinical severity in patients with COVID-19. This may provide the basis for effective therapeutic approaches in the coming years.

## Materials and Methods

### Study participants

The present study was conducted at the Medical Biochemistry Department, internal medicine department, faculty of medicine and Zoology Department, Faculty of Science, Mansoura University, Egypt. The present study comprised 40 patients with COVID-19 and 40 age-matched and sex-matched apparently healthy controls recruited between September 2021 and March 2022. This study was approved by the Mansoura University ethics committee (Institutional Research Board; R.20.10.1) and all patients provided signed informed consent.

All patients were admitted to the ICU with severe pulmonary manifestations around 10 days from the onset of the initial symptoms. The study participants were assigned to one of two groups. Group 1 comprising 40 patients (22 males and 18 females) with severe COVID-19 infection who were admitted to the Intensive Care Unit (ICU) at the time of the study and may have received remdesivir and/or inotropes. All patients had a confirmed diagnosis of COVID-19 based on their clinical profile and a positive PCR test. Group 2 comprised 40 healthy controls who had no previous history of COVID-19 infection, chronic pulmonary disease, or autoimmune disease. Patients with mild respiratory symptoms or pregnant women with COVID-19 were excluded from the present study.

### Blood sample collection

The sampling was done at one time point in patients with severe COVID-19, within 48 h of ICU admission. In healthy volunteers, blood samples were obtained similarly at one point-time. Venesection was performed for all participants to obtain 7 ml of whole blood that was divided into two collection tubes; 4 ml of blood were collected in EDTA-containing tubes for isolation of peripheral blood mononuclear cells and the remaining 3 ml were collected in plain tubes and used for serum biochemical analyses.

### Biochemical analysis

Serum levels of melatonin (MLT; Catalog # EH3344) and malondialdehyde (MDA; Catalog # EU2577) were measured using ELISA kits purchased from Fine Test, Wuhan Biotech, China according to the manufacturer’s instructions. TAC was measured using ELISA kits obtained from Raybiotech Norcross, GA, USA, (Catalog # ELH-ITAC-1) in accordance with the manufacturer’s instructions.

### Isolation of peripheral blood mononuclear cells

Whole blood (4 ml) was collected from each subject in EDTA-containing collection tubes. Isolation of peripheral mononuclear blood cells (PBMCs), T cells, B cells, and monocytes was performed using Histopaque-1077 (Sigma-Aldrich) Ficoll density-gradient centrifugation according to manufacturer’s instructions.

### RNA extraction and cDNA synthesis

Total RNA including lncRNA was extracted from PBMCs using TRIzol reagent (Zymo Research, Irvine, CA). A NanoDrop 2000 spectrophotometer (Thermo. Fischer Scientific, Waltham, MA) was used to quantitate RNA. Total RNA samples were stored at − 80 °C until further use. SensiFAST cDNA Synthesis kits (Bioline, Memphis, TN) were used for cDNA synthesis according to manufacturer’s instructions. Reverse transcription was performed in reactions with a final volume of 20 ml.

### Real-time PCR

ACE2 expression levels were measured using Hera plus SYBR Green qPCR kits (Willow Fort, Birmingham, UK) with GAPDH used as an internal control according to the manufacturer’s protocol. Fold change was calculated using the comparative threshold cycle method [2^−ΔΔCt^] for relative quantification normalized to an endogenous control [22].

The following primers were used for ACE2 and GAPDH genes:

**Table:** 

Gene	Forward	Reverse
ACE-2	5'-TCCATTGGTCTTCTGTCACCCG-3'	5'-AGACCATCCACCTCCACTTCTC-3'
GAPDH	5'-ACAGTCAGCCGCATCTTCTT-3'	5'-GACAAGCTTCCCGTTCTCAG-3'

Real-time PCR was performed in 20 ml reaction mixtures using a 7500 Real-time PCR System (Applied Biosystems) with the following conditions: 95 °C for 10 min followed by 40 cycles at 95 °C for 15 s and a final step of 60 °C for 60 s.

### Statistical analyses

Data were stored and analyzed using IBM-SPSS software (IBM Corp. Released 2019. IBM-SPSS Statistics for Windows, Version 26.0. Armonk, NY: IBM Corp) and MedCalc® Statistical Software version 20 (MedCalc Software Ltd, Ostend, Belgium, 2021). Qualitative data are presented as n (%). Quantitative data were initially tested for normality using Shapiro–Wilk’s test with data considered normally distributed if P > 0.050. The presence of significant outliers was tested by inspecting boxplots. Quantitative data were expressed as the mean and standard deviation or median and interquartile range (Q1–Q3). Qualitative data were compared between groups using the Chi-Square (χ^2^) test. Quantitative data were compared between two groups using the independent-samples t-test (normally distributed) or Mann–Whitney U-test (non-normally distributed). The one-sample Wilcoxon signed ranks test was used to compare non-normally distributed quantitative data against a hypothetical median. Spearman’s correlation was used to assess the direction and strength of association between two non-normally distributed continuous variables. The strength of association was considered low, medium, or large for correlation coefficients < 0.3, 0.3–0.5, and > 0.5, respectively. Point biserial correlation was used to assess the association between a dichotomous variable and a continuous variable. Receiver operating characteristic (ROC) curves were used to categorize continuous variables into two categories based on a cutoff value with the area under the curve (AUC) reported. Diagnostic accuracy was considered excellent, very good, good, sufficient, or bad when the AUC was 0.9–1.0, 0.8–0.9, 0.7–0.8, 0.6–0.7, or 0.5–0.6, respectively. P-values ≤ 0.05 were considered statistically significant. Charts were used to graphically present the results as appropriate.

## Results

The present study comprised 40 confirmed cases of COVID-19 including 22 males and 18 females. Patients with COVID-19 had a mean age of 60.7 ± 9.29 years. The present study also included 40 age-matched and sex-matched healthy control subjects (26 males and 14 females) with a mean age of 59.6 ± 9.31 years. There was no significant difference in sex or age between the two groups (P < 0.361 and P < 0.590, respectively).

**Fig. 1:**
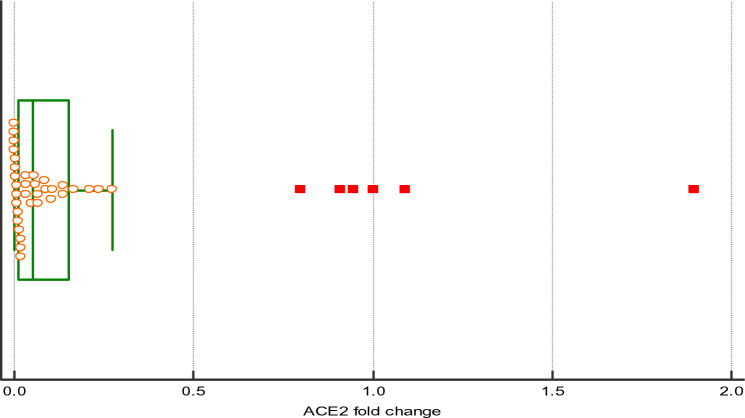
ACE2 expression levels. Median ACE2 (FC), 0.05; 95% CI, 0.0.017 to 0.098; Hodges–Lehmann location estimator, 0.0766; P < 0.001)

ACE2 mRNA expression was compared between the two study groups, one-sample Wilcoxon signed ranks test also demonstrating significantly lower expression levels of ACE2 in COVID-19 patients compared to a hypothesized value (1.0) for controls. Median ACE2 fold change (FC) = 0.05, 95% CI for the median = 0.0.017 to 0.098). This was statistically significantly lower than the normal control value of 1.0 (Hodges-Lehmann location estimator = 0.0766, P < 0.001) (Fig. 1).

Serum levels of oxidative stress markers (MDA, MLT, and TAC) were compared between the two groups. Serum levels of TAC and MLT were significantly lower in COVID-19 patients compared to controls (P > 0.001). On the other hand, patients with COVID-19 had significantly higher serum levels of MDA (P > 0.001) compared to healthy controls (Table 1).

**Table 1:** Levels of MLT, TAC and MDA in both groups

Biomarker	Control	COVID-19	Hodges–Lehmann median difference	P-value
MLT (pg/ml)	141 (122.3–161.3)	62.8 (31.8–111.3)	75.5 (53.2–91.2)	< 0.001
TAC (mmol/L)	1.63 (1.35–1.84)	0.90 (0.43–1.36)	0.69 (0.45–0.93)	< 0.001
MDA (nmol/ml)	0.38 (0.28–0.48)	2.29 (0.76–10.8)	− 1.84 (− 7.06 to − 0.71)	< 0.001

Data are presented as the median with interquartile range. P-values represent comparisons using the Mann–Whitney U-test

The correlation of ACE2 mRNA and serum levels of oxidative markers with clinicopathological data was next examined. These correlations aimed to assess the prognostic validity of the studied markers in predicting the disease severity and outcomes. Therefore, therapeutic approaches targeting oxidative stress markers may curtail the systemic consequences of severe SARS-COV2 infection. The data presented in (Table 2) demonstrates a statistically significant negative correlation between serum MDA levels and both diastolic blood pressure (DBP) and Glasgow coma scale (GCS) scores. Serum MLT levels were significantly and positively correlated with DBP, MAP, and respiratory rate. Serum levels of TAC were positively correlated with GCS scores.

**Table 2:** Correlation between the four studied biomarkers and clinical data in COVID-19 cases (n = 40)

Clinical data	TAC		MDA		MLT		ACE2	
	r_s_	P-value	r_s_	P-value	r_s_	p-value	r_s_	P-value
Age (years)	− 0.097	0.552	0.153	0.346	− 0.111	0.495	− 0.150	0.374
Admission duration (days)	− 0.307	0.054	0.170	0.293	0.046	0.776	− 0.171	0.292
Systolic blood pressure (mmHg)	− 0.064	0.697	− 0.019	0.907	0.170	0.300	0.060	0.715
Diastolic blood pressure (mmHg)	0.238	0.144	− 0.335^*^	0.037	0.490**	0.002	0.068	0.681
Mean arterial blood pressure (mmHg)	0.066	0.690	− 0.154	0.350	0.330*	0.040	0.074	0.652
Heart rate	0.176	0.284	− 0.178	0.277	0.176	0.285	0.099	− 0.050
Temperature	0.099	0.542	− 0.184	0.255	0.230	0.154	− 0.050	0.757
Respiratory rate	0.236	0.161	− 0.300	0.071	0.352*	0.032	0.140	0.408
Glasgow coma scale (GCS)	0.365	0.042	− 0.404	0.020	0.294	0.096	0.281	0.113

*r*_*s*_ Spearman’s correlation coefficient

**P < 0.01

*P < 0.05 (two-tailed)

A statistically significant negative correlation was observed between serum TAC and creatinine levels, between ACE2 mRNA expression and total bilirubin levels, and between serum MDA and potassium levels (Table 3). A statistically significant and positive correlation was observed between serum MLT and potassium levels. ACE2 mRNA expression levels did not correlate with any complete blood count (CBC) parameter.

**Table 3:** Correlations between biochemical parameters and ACE2 mRNA expression, TAC, MDA, and MLT levels in COVID-19 cases (n = 40)

Lab test	TAC		MDA		MLT		ACE2	
	r_s_	P-value	r_s_	P-value	r_s_	P-value	r_s_	P-value
Serum sodium	0.007	0.966	− 0.042	0.800	0.168	0.306	− 0.0285	0.079
Serum potassium	0.244	0.134	− 0.344	0.032*	0.451	0.004**	0.201	0.219
Random blood glucose (RBG)	− 0.102	0.532	0.074	0.649	0.032	0.846	0.170	0.295
ALT (IU/L)	0.309	0.059	0.248	0.133	0.094	0.576	0.189	0.259
AST (IU/L)	− 0.113	0.512	0.038	0.420	0.048	0.781	− 0.306	0.07
Serum albumin	− 0.116	0.581	0.130	0.429	0.160	0.331	0.252	0.122
Serum total bilirubin	0.153	0.545	0.164	0.515	0.215	0.391	− 0.506	0.032*
Serum creatinine	− 0.331	0.037*	0.264	0.099	0.169	0.296	0.098	0.549
Serum LDH	0.47	0.370	0.140	0.556	0.347	0.134	0.186	0.432
C-reactive protein	− 0.031	0.876	0.161	0.422	0.158	0.432	− 0.208	0.297
RBCs count	− 0.214	0.185	0.160	0.325	− 0.100	0.539	− 0.216	0.181
Hemoglobin	− 0.202	0.212	0.179	0.269	− 0.142	0.381	− 0.080	0.623
Hematocrit	− 0.169	0.298	0.151	0.352	− 0.106	0.513	− 0.147	0.365
MCV	0.025	0.876	− 0.012	0.944	0.042	0.798	0.049	0.763
MCH	− 0.038	0.815	0.027	0.871	− 0.022	0.894	0.168	0.299
MCHC (g/dl)	− 0.164	0.326	0.192	0.247	− 0.293	0.074	0.295	0.072
RDW (%)	0.000	0.999	0.089	0.623	− 0.124	0.492	− 0.160	0.374
Platelet count	− 0.088	0.590	0.054	0.739	− 0.064	0.696	0.158	0.330
Mean platelet volume	0.509^**^	**0.008**	− 0.344	0.086	0.121	0.556	0.205	0.316
Total WBC count	0.040	0.808	− 0.028	0.862	0.073	0.656	− 0.193	0.394
Neutrophil count	0.203	0.281	− 0.239	0.203	0.40	0.263	− 0.043	0.822
Lymphocyte count	0.035	0.829	− 0.042	0.797	− 0.002	0.988	− 0.251	0.118
Monocyte count	0.407	0.317	− 0.419	0.301	0.072	0.866	0.096	0.821
Basophil count	0.327	0.429	− 0.436	0.280	0.546	0.162	0.109	0.797
Eosinophil count	0.164	0.699	− 0.436	0.280	0.409	0.314	− 0.600	0.116
INR	0.116	0.526	− 0.183	0.316	0.272	0.133	0.025	0.894
pH	0.257	0.110	− 0.086	0.596	− 0.037	0.821	0.101	0.533
HCO_3_	0.237	0.152	− 0.204	0.218	0.172	0.301	0.016	0.926
PaO_2_	0.063	0.705	− 0.090	0.586	0.154	0.350	− 0.128	0.439
PaCO_2_	0.0.085	0.603	− 0.215	0.183	0.289	0.071	0.014	0.933
D-dimer	− 0.132	0.416	0.096	0.554	0.030	0.853	− 0.083	0.612

*r*_*s*_ Spearman’s correlation coefficient

**P < 0.01; *P < 0.05 (two-tailed)

Moreover, serum TAC levels were significantly and positively correlated with mean platelet volume (P = 0.008). ACE2 mRNA expression levels and the studied redox state markers were not correlated with arterial blood gas (ABG) parameters.

Patients with COVID-19 who were treated with remdesivir had significantly lower serum levels of MLT (P = 0.025) compared to the control group (Table 4). Serum MLT levels were lower in patients treated with inotropes (median serum MLT level, 22.7) compared to patients not treated with inotropes (median serum MLT level, 74.4).

**Table 4:** Correlations between categorical data and ACE2 mRNA expression, serum TAC, serum MDA, and serum MLT levels in COVID-19 cases (n = 40)

Categorical variable	TAC		MDA		MLT		ACE2	
	r_pb_	P-value	r_pb_	P-value	r_pb_	P-value	r_pb_	P-value
Use of remdesivir	− 0.200	0.216	0.220	0.173	− 0.355	0.025	0.229	0.156
ICU admission	− 0.068	0.677	0.109	0.505	0.139	0.393	− 0.083	0.609
Diabetes	− 0.219	0.174	0.113	0.486	− 0.031	0.851	0.197	0.223
Hypertension	− 0.174	0.284	0.235	0.145	0.027	0.867	0.052	0.751
Coronary artery disease	0.076	0.641	0.045	0.784	0.031	0.850	− 0.103	0.526
Chroninc kidney disease on haemodialysis (CKD-HD)	− 0.213	0.187	0.274	0.087	− 0.170	0.293	− 0.110	0.500
Use of inotropes	0.014	0.929	− 0.003	0.986	− 0.373	0.018	− 0.117	0.471
Mechanical ventilation	0.010	0.951	0.087	0.594	0.098	0.547	− 0.024	0.884

*r*_*pb*_ point biserial correlation coefficient, *CKD-HD* chronic kidney disease on maintenance hemodialysis

**Fig. 2:**
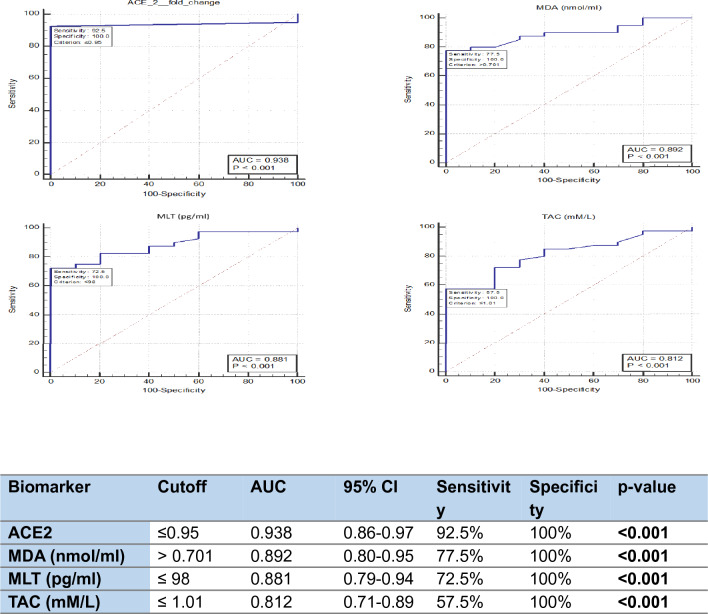
Diagnostic performance of serum ACE2 and oxidative markers. ROC curve analysis of serum ACE2 and oxidative markers for the ability to discriminate between patients with COVID-19 (n = 40) and healthy controls (n = 40). AUC, area under the ROC curve; CI, confidence interval; SE, standard error

ROC curve analysis was performed to assess the diagnostic performance of ACE2 mRNA expression levels and the studied oxidative markers for COVID-19 infection and demonstrated ACE2 mRNA expression levels and the studied oxidative markers could discriminate COVID-19 patients from healthy controls (Fig. 2).

## Discussion

Oxidative stress has a key role in the pathogenesis of COVID-19 due the development of an imbalance between the production of ROS and the body’s antioxidant capacity [23]. In COVID-19, the main target of SARS-CoV-2 is ACE2 receptor, a critical enzyme of the renin–angiotensin–aldosterone system (RAAS), which promotes oxidative stress via stimulating NADPH Oxidase, an action principally mediated by Ang II [24]. SARS-CoV-2 infection results in accumulation of AngII, and ROS causing oxidative stress and cell damage[25]. A wide range of viral diseases, including HIV 1, the viral hepatitis B, C, and D, herpes viruses, and respiratory viruses such as coronaviruses, are known to contribute to oxidative stress [26]. Accordingly, greater understanding of the molecular mechanisms underlying the contribution of oxidative stress to the pathogenesis of COVID-19 may facilitate the development of novel therapies. The present study evaluated the expression levels of angiotensin converting enzyme 2 (ACE2) and redox state markers (TAC, MLT, and MDA) in patients with severe COVID-19 admitted to the ICU with pulmonary manifestations. Several studies proposed the association between the studied redox state markers (TAC, MLT, and MDA) and the pathogenesis of COVID-19. MDA is a known indicator of oxidative stress, being an indicator of free radicals-induced oxidative damage in COVID-19 patients [13, 27].

Our results demonstrate that ACE2 mRNA expression levels were significantly lower in patients with severe COVID-19 compared to healthy controls. ROC curve analysis evaluated the diagnostic performance of markers in COVID-19 demonstrated the highest diagnostic accuracy among markers of oxidative stress (AUC = 0.938%), with a sensitivity of 92.5% and specificity of 100% in discriminating COVID-19 cases from control subjects. ACE2 is the primary receptor for SARS-CoV-2 entry into host cells [28]. Attachment of viral spike proteins to ACE2 receptors at the cell membrane facilitates entry of SARS-CoV-2, the causal agent of COVID-19 (26). Accordingly, susceptibility to SARS-CoV-2 infection may be linked to expression levels of ACE2 receptor at the endothelium following viral exposure [29]. The levels of plasma ACE2 have been assessed in the early phase of SARS-COV-2 infection, a significant increase in soluble ACE2 has been reported, principally in severe cases suggesting an excessive shedding of ACE2 during the early phase of SARS-CoV-2 infection [30]. However, Contradictory reports on the correlation between ACE2 expression levels and disease severity and outcomes are being available [31, 32]. The results of the present study demonstrate that patients with severe COVID-19 at the late phase of infection exhibit lower ACE2 mRNA expression. This finding corroborates previous evidence suggesting that the SARS-CoV-2 virus downregulates ACE2 expression following cell entrance [33]. Several studies have indicated that downregulation of membrane-bound ACE2 may induce dysfunction of the immune system and contribute to poor outcomes in patients with COVID-19 [34, 35]. SARS-CoV-2 may induce downregulation of ACE2 by a number of mechanisms including: (1) decreased ACE2 receptor expression due to immune dysfunction; (2) enhanced shedding of membrane-bound ACE2; and (3) endocytosis of ACE2 receptor with SARS-CoV-2 [34]. Downregulation of ACE2 has been shown to alter the ratio of ACE to ACE2 in many pathological conditions[36]. Accordingly, high ACE levels may suggest low ACE2 levels and vice versa [34].

The results of the present study also demonstrate a negative correlation between ACE2 mRNA expression and serum bilirubin levels. Previous studies have reported that hyperbilirubinemia is an indicator of liver injury in patients with severe COVID-19 [37, 38]. The findings of the present study indicate that enhancing and maintaining ACE2 expression may represent a potential therapeutic option for severe COVID-19.

Furthermore, serum MLT and TAC levels were significantly lower while serum MDA levels were significantly higher in patients with severe COVID-19 compared to healthy controls. We believe this to be the first study to demonstrate low serum levels of MLT in patients with COVID-19. MLT is a major component of the antioxidative defense system against infection due to its ability to scavenge free radicals and stimulate a number of antioxidant enzymes [39]. TAC is a known predictor of disease severity in several diseases. Previous studies have reported that TAC is reduced in patients with COVID-19 compared to healthy controls [40, 41]. In the present study, TAC was positively correlated with GCS scores, and patients with less severe disease had higher serum TAC levels. Moreover, TAC was negatively correlated with serum creatinine levels in the COVID-19 group, indicating patients with higher TAC levels had more stable kidney function. However, TAC has been reported to be correlated with disease severity and negative outcomes in many other clinical diseases[42–44]. This finding may represent intense protective mechanisms against overwhelming inflammation. Accordingly, the present study found a positive correlation between TAC and mean platelet volume (MPV), which is considered a significant predictor and prognostic biomarker of inflammation and oxidative stress[45–47]. Of note, previous studies have reported MPV predicts severe COVID-19 [48–50].

MLT has remarkable antioxidant and anti-inflammatory properties may protect against the proinflammatory cytokine storm and neutralize free radicals, thereby maintaining cellular integrity and minimizing lung injury[51]. In the absence of acetyl-coenzyme A, mitochondrial MLT is no longer available to reduce the inflammatory response or neutralize generated ROS, thereby contributing to the massive pulmonary injury in patients with severe COVID-19 [52]. This is in line with the findings of the present study, which found that COVID-19 patients had lower serum MLT levels than healthy controls, which we believe to be a novel finding.

MDA is a byproduct of the cyclo-oxygenase reaction in prostaglandin metabolism [53], with serum MDA levels reported to be positively correlated with COVID-19 severity in earlier studies [40, 54]. In the present study, serum MDA levels were negatively correlated with GCS and DBP, indicating patients with higher serum MDA levels were more likely to have severe disease and decreased consciousness levels. Furthermore, lower serum potassium levels were observed in patients with higher serum MDA levels and lower serum MLT levels.

Previous studies have reported that MLT plays a vital role in regulating the cardiovascular system and has a hypotensive effect in mammals[55, 56]. However, a positive correlation was observed between serum MLT levels and both systolic and diastolic blood pressure (DBP) in the present study, with a statistically significant correlation observed between serum MLT levels and DBP.

Although elevated levels of D-dimer have been previously shown as a predictor of COVID-19 activity [30, 57], the present study did not find any significant correlation between D-dimer on one hand with ACE2 expression and the studied oxidative stress markers on the other hand.

Further studies are required to validate this observation. However, the patients included in the present study had a different clinical profile to typical patients with cardiovascular disease. Moreover, the present study found that patients with lower serum MLT levels were more likely to require treatment with remdesivir and inotropes, which are more frequently required in patients with more severe disease.

Among the main strengths of the present study is that it focused on patients with severe COVID-19 at a late phase of the disease, included assays of a variety of oxidative stress markers by different assessment methods, considered extensive correlations to the clinic-laboratory-pathologic criteria which may provide a basis for targeting COVID-19 with new therapeutic approaches.

## Conclusion

In conclusion, the results of the present study demonstrate that increased levels of oxidative stress markers, decreased levels of antioxidant indicators, and lower ACE2 expression levels are correlated with the disease severity and poor outcomes in hospitalized patients with COVID-19. These findings indicate ACE2, and oxidative stress markers may represent therapeutic targets for severe COVID-19.

## Data Availability

All data generated or analyzed during this study are included in this published article.
